# Predicting Textbook Oncological Outcomes with Machine Learning: A Retrospective Review of Patients Who Underwent Resection for Malignant Tumours

**DOI:** 10.3390/jcm15218527

**Published:** 2026-11-02

**Authors:** Meet Patel, Jonathan Ben Daniel, Nazim Bhimani, Joshua G. Kovoor, Arjun Fadia, Kai Brown, Anthony R. Glover, Thomas J. Hugh

**Affiliations:** 1 Faculty of Medicine and Health, The University of Sydney, Camperdown, NSW 2050, Australianazim.bhimani@sydney.edu.au (N.B.); kai.brown@sydney.edu.au (K.B.); anthony.glover@sydney.edu.au (A.R.G.); tom.hugh@sydney.edu.au (T.J.H.); 2 Upper Gastrointestinal Surgical Unit, Royal North Shore Hospital, St Leonards, NSW 2065, Australia; 3 Faculty of Health and Medical Sciences, University of Adelaide, Adelaide, SA 5000, Australia

**Keywords:** hepatectomy, outcomes, oncology, machine learning, textbook

## Abstract

**Introduction**: Textbook oncological outcome (TOO) is a composite measure used to assess the quality of care in surgical oncology. Specifically, in liver surgery, it assesses several domains including intraoperative events, postoperative recovery, liver-specific complications, early mortality, and resection margins. Historically, one-third of patients undergoing liver resection have had a non-ideal outcome that has been difficult to predict. The aim of this study is to predict the risk of achieving textbook oncological outcome (TOO) using machine learning (ML) models for patients undergoing liver resection for malignant tumours. **Methods**: A retrospective cohort study of patients undergoing liver resection for malignant tumours was undertaken between 1999 and 2023 from a prospectively collected database. ML models were developed using preoperative variables as candidate predictors of TOO. Feature selection was performed using stability selection with LASSO regularisation. Model performance was evaluated using discrimination and calibration metrics. Model interpretability was assessed using SHapley Additive exPlanations. Predicted probabilities were stratified into tertiles to form risk groups (low, moderate, and high). **Results**: From the training cohort (n = 583), the RF model demonstrated the highest discrimination (AUROC = 0.813, 95% confidence interval [CI]= 0.731–0.877) for predicting TOO in the test cohort (n = 146). Post-recalibration, the Brier score was 0.162, calibration intercept was 0.06 and calibration slope was 0.92. The top three features were the extent of the resection, the site of the tumour, and the size of the largest tumour. TOO rates decreased in low-, moderate-, and high-risk groups (91%, 80% and 38% respectively, *p* < 0.001). **Conclusions**: An ML-based model for predicting TOO following liver resection demonstrated good discrimination and calibration metrics after recalibration. Risk stratification showed a clear gradient in TOO achievement, supporting its potential use in preoperative risk assessment and patient counselling. The current exploratory nature of this study necessitates further external validation before clinical application.

## 1. Introduction

Textbook oncological outcome (TOO) is a composite measure used to assess the quality of surgical care and has been applied in numerous surgical specialties and procedures. It is based on the principle of “all or nothing”, i.e., success is measured only if all predefined criteria are met. Specifically, in liver surgery, a consensus definition has been proposed by Gorgec et al. (2023) [1]. Here, TOO was defined as the absence of seven criteria, as follows: grade ≥ 2 intraoperative incidents, 90-day postoperative complications of Clavien–Dindo III or higher, 90-day readmission due to surgery-related complications of Clavien–Dindo Grade 3 or higher, postoperative bile leakage of grades B and C, postoperative liver failure of grades B and C, in-hospital or 90-day mortality, and R1 or R2 resection margins [1]. The purpose of this definition was to provide an appropriate metric that captures the patient journey across a number of domains (intraoperative incidents, general postoperative complications, liver surgery-related postoperative complications, mortality, oncological resection margin), i.e., ensuring that the surgery will go without a hitch. They also proposed an extended definition of TOO that included discharge within a specific time postoperatively based on the extent of the resection, but consensus was not reached [1]. Following a review of the literature, the median rate of achieving TOO in hepatic procedures was 62% (interquartile range (IQR) 48.0−69.0%) compared to 45.0% (IQR 30.5−59.0%) for pancreatic procedures [2]. The fact that almost one-third of patients who undergo liver surgery do not have an ideal postoperative recovery suggests that the identification of high-risk patients may improve patient selection and assist with the informed consent process.

TOO provides an assessment of the quality of healthcare and can provide a benchmark for institutions to identify areas of improvement across different domains of care. The aim is to move beyond the traditional metrics of surgical care, including morbidity, mortality, and postoperative length of stay (LOS), and instead, to have a single, unifying metric that is easily interpreted and clinically useful. Not only has TOO been helpful in quality assessment, but long-term outcome data analyses have also shown that achieving TOO after hepatic resection is associated with improved overall survival and disease-free survival in patients with hepatocellular carcinoma, cholangiocarcinoma, and colorectal liver metastases [2,3,4,5,6]. Additionally, McGarry et al. (2025) demonstrated that in patients who underwent resection of colorectal liver metastases, the attainment of TOO was associated with higher patient-reported quality of life than non-attainment of TOO [6]. This is not surprising given that the definition of TOO includes major complications, readmissions, and reoperations, all of which are already known to impact quality of life, at least in the short-term. However, TOO does not currently incorporate patient-reported outcome measures (PROMs), functional recovery, or time-to-adjuvant therapy, which are all recognised as important quality dimensions. Regardless, TOO is a pragmatic composite measure for quality benchmarking in surgical oncology, and the value for preoperative risk communication to patients has not been fully investigated. Identifying and preparing patients who are at higher risk for not achieving TOO in the preoperative setting may assist with the consent process and better equip patients and caregivers for all possible postoperative recovery outcomes.

Previous studies have attempted to rationalise factors associated with not achieving TOO to assist with patient selection and counselling. A complex interplay between patient, disease and technical factors has been identified in predicting TOO [3,4,7,8,9]. Recently, machine learning (ML) has emerged as a potential tool for clinical outcome prediction, diagnosis, treatment, and optimal decision-making. This is a subset of artificial intelligence, where an algorithm can be constructed to develop an optimised function based on data and the specific task [10]. By using a large prospectively collected database of patients who underwent liver resection, we hypothesised that ML would be able to develop a predictive risk score associated with TOO in patients with malignant disease. The aim of this paper is to develop a model associated with TOO in patients undergoing liver resection for malignant disease using ML algorithms to be used in future prospective research.

## 2. Methods and Materials

### 2.1. Ethics and Consent

Ethics approval was granted by the Northern Sydney Local Health District Human Research Ethics Committee in accordance with the National Statement on Ethical Conduct in Human Research, 2023 (Approval no: 2024/ETH00274 V2). Prospective data collection for the original database was conducted under ethics approval granted by the Northern Sydney Local Health District Human Research Ethics Committee (Approval no: 2019/ETH12206). This study conforms to the Transparent Reporting of a multivariable prediction model for Individual Prognosis Or Diagnosis, Artificial Intelligence (TRIPOD+AI) checklist, where appropriate, for reporting on the development of clinical prediction models (Supplementary Material Table S1) [11].

### 2.2. Participants

A retrospective cohort study of patients who underwent liver resection at Royal North Shore Hospital and North Shore Private Hospital (Sydney, Australia) under the care of a single, hepato-biliary surgeon (TJH) was undertaken. Data were prospectively collected and maintained in a database of consecutively completed liver resections. Inclusion criteria were patients who underwent liver resection from 1999 to 2023, patients aged ≥18 years, patients who underwent resection for malignant tumours, and resection completed with curative intent. Patients who underwent a palliative resection, resection for benign disease, diagnostic laparoscopy only, liver biopsies only, or procedures that were abandoned because of unexpected metastatic disease were excluded from the study.

TOO was defined as the absence of seven criteria as described in the consensus statement by Gorgec et al. (2023) [1].

### 2.3. Operative Approach

Intraoperative ultrasound was used to confirm the tumour location and relationships with major vascular and biliary structures. Parenchymal transection was performed using the Cavitron Ultrasonic Surgical Aspirator (CUSA) dissector (Integra LifeSciences Corp, Princeton, NJ, USA) under low central venous pressure conditions, and with intermittent inflow occlusion.

### 2.4. Machine Learning Model Development

Data were imported from the database and processed using a structured pipeline in Python (version 3.11). Preoperative variables were classified as continuous or nominal. Definitions for the variables are outlined in Supplementary Material Table S2a,b. Era-based adjustment was not included as it was shown previously that TOO rates did not change across era in this cohort in a previously published paper by this group Patel et al. (2026) [12]. Median imputation was used for missing continuous variables. Missing categorical variables were imputed as ‘missing’ and treated as a separate category during one-hot encoding. Median imputation was retained for continuous variables because it allows a consistent preprocessing pipeline across algorithms and is resistant to extreme values. Missing categorical values were retained as a separate level to avoid complete-case deletion and loss of sample size. To prevent data leakage, all intraoperative and postoperative variables were excluded from preprocessing and model training. Constant features, defined as those in which a single value accounted for more than 97% of observations, were removed prior to modelling to simplify the feature space. After preprocessing, the data were randomly divided into training and test sets using stratified random sampling implemented via the train_test_split() function from the scikit-learn library, with an 80:20 ratio and fixed random seed for reproducibility. Further feature reduction was performed using a stability-selection framework based on LASSO regularisation to identify consistent informative variables in the training set. Features retained in ≥90% of resampling iterations were considered stable and were carried forward for modelling.

Six machine learning classifiers were developed and tuned within nested cross-validation: logistic regression (LR), random forest (RF), gradient boosting (GB), histogram gradient boosting (HGB), Extreme Gradient Boosting (XGB), and Light Gradient Boosting Machine (LGBM). Logistic regression provides an interpretable linear baseline, while tree-based ensemble methods (RF, GB, HGB, XGB, LGBM) capture nonlinear interactions through combinations of decision trees [13,14,15]. Gradient boosting methods (XGB, LGBM) sequentially improve predictions, whereas RF aggregates independent trees [15]. Each model underwent randomised hyperparameter search using RandomizedSearchCV function from sklearn (100–150 iterations to optimize AUROC) evaluated across 10 folds with 3 repetitions, giving 30 stratified validation folds in total. To estimate model performance during development while separating hyperparameter optimisation from performance assessment, an additional nested cross-validation procedure was performed using a 3-fold outer loop and 3-fold inner loop. The inner loop was used for model tuning, while the outer loop provided an unbiased estimate of cross-validated discrimination. It is noted that for feature selection, bootstrapped LASSO was used, which involves a linear model that can introduce bias in tree-based models (e.g., RF, XGB, LGBM) by under-selecting features that contribute via nonlinear or interaction effects captured by tree-based models. As a result, a sensitivity analysis was completed on all features for RF, XGB and LGBM.

The ML pipeline development of a model uses a single preprocessing pipeline across models, which included median imputation for continuous variables. This was completed to ensure comparability across linear and tree-based models. However, gradient-boosted trees (e.g., XGB/LGBM) can natively handle missing values, meaning median imputation may therefore attenuate the signal of variables with informative missingness, potentially reducing their contributions.

After hyperparameter tuning, all models were completely trained on the full training set and evaluated on the test set. Model performance was compared through the discrimination of each model using the c-statistic (area under the receiver operating characteristic curve [AUROC]). Harrell’s bootstrap validation was used to determine the optimism-corrected training AUROC. The DeLong test was used to compare the models with the two highest observed AUROC. SHapley Additive exPlanations values were computed on the training data using shap.TreeExplainer for tree-based models. SHAP values were calculated on the model’s native additive output scale. The SHAP value for each feature indicates its impact on a specific prediction. Positive SHAP values indicate contributions toward a higher predicted likelihood of TOO relative to the model-specific baseline output, whereas negative SHAP values indicate contributions toward a lower predicted likelihood. Model performance was assessed using discrimination on the test cohort, with AUROC used as the primary performance metric. Quantitative SHAP analysis was used to assess the relative contribution of included variables and to identify variables with negligible or marginal contributions.

Calibration in the independent test group was evaluated globally using the Brier score. The calibration intercept and slope were estimated by fitting a logistic regression model in the test cohort, with the observed outcome as the dependent variable and the logit-transformed predicted probability as the independent variable. A calibration curve was constructed by grouping predicted probabilities into deciles and plotting the mean predicted probability against the observed event rate within each decile. Recalibration was performed by fitting a logistic regression model in the training cohort with the observed outcome as the dependent variable and the logit-transformed predicted probability as the predictor. Logistic recalibration applies a monotonic transformation to the original predicted probabilities. Provided the fitted slope is positive, this transformation preserves their rank ordering and therefore leaves the AUROC unchanged, while altering the absolute probabilities and potentially improving calibration and the Brier score. The resulting calibration parameters were then applied to the independent test cohort predictions. Discrimination and calibration metrics were reassessed following recalibration. Decision curve analysis was performed in the test cohort using the predicted probability of non-achievement of TOO. The analysis was undertaken as an exploratory assessment of whether the model-guided identification of patients at increased risk of non-TOO achievement could inform escalation to an enhanced preoperative risk management pathway such as additional MDT review or more detailed risk counselling. In this context, ‘treat all’ refers to enhanced risk management pathways for all patients, whereas ‘treat none’ refers to usual care without model-triggered escalation. There is no clinically validated threshold for non-TOO risk, and as such threshold probabilities were evaluated across the full range for exploratory purposes and were not interpreted as recommendations for clinical intervention.

Patients in the test group were stratified into three risk groups (low operative risk, moderate operative risk, high operative risk), using tertiles from the training cohort as cut-off scores post-recalibration to improve interpretability. In this context, ‘operative risk’ refers specifically to the predicted risk of non-achievement of TOO. Tertiles were used as an exploratory, distribution-based approach to assess whether increasing predicted risk was associated with an ordered gradient in observed TOO rates while maintaining adequate sample size within each group.

### 2.5. Statistical Analysis

Continuous baseline variables that were normally distributed were presented as mean with standard deviation (SD), while non-normally distributed continuous variables were reported as median and IQR. Categorical variables were presented as frequencies with percentages. Chi-square tests were used to compare proportions between groups for categorical variables. For continuous variables, groups were compared using an independent-samples *t*-test for normally distributed data, and the Mann–Whitney U test was used for continuous data that were not normally distributed. Statistical analysis was performed using R 4.5.1 (The R Foundation for Statistical Computing, 2025) and Python (version 3.11).

## 3. Results

A total of 729 patients who underwent liver resection were identified. Baseline characteristics are summarised in Table 1 and Table 2. The mean age of the cohort was 64.5 years and 56.9% of patients were male. The most common indication for resection was colorectal liver metastases (60.5%), followed by hepatocellular carcinoma (12.1%) and cholangiocarcinoma (9.6%). Most patients had open resections (89.6%).

Postoperative clinical outcomes are summarised in Table 2. In total, 68.3% of patients achieved a TOO. The most common reason for failure to achieve TOO was due to R1/R2 resection margins (21%), followed by postoperative complications of Clavien-Dindo grade ≥ 3a (13%). There was relatively little missing data, with 1.9% of the cohort missing data on the diameter of the largest tumour and 2.1% of the cohort missing data on the number of tumours (Table 2).

### 3.1. Training, Testing and Validation of Algorithms

Preoperative variables were selected based on the exclusion of constant variables and LASSO regularisation. The complete list of the variables included in and excluded from the model development is found in the Supplementary Material Table S2a,b.

**Figure 1:**
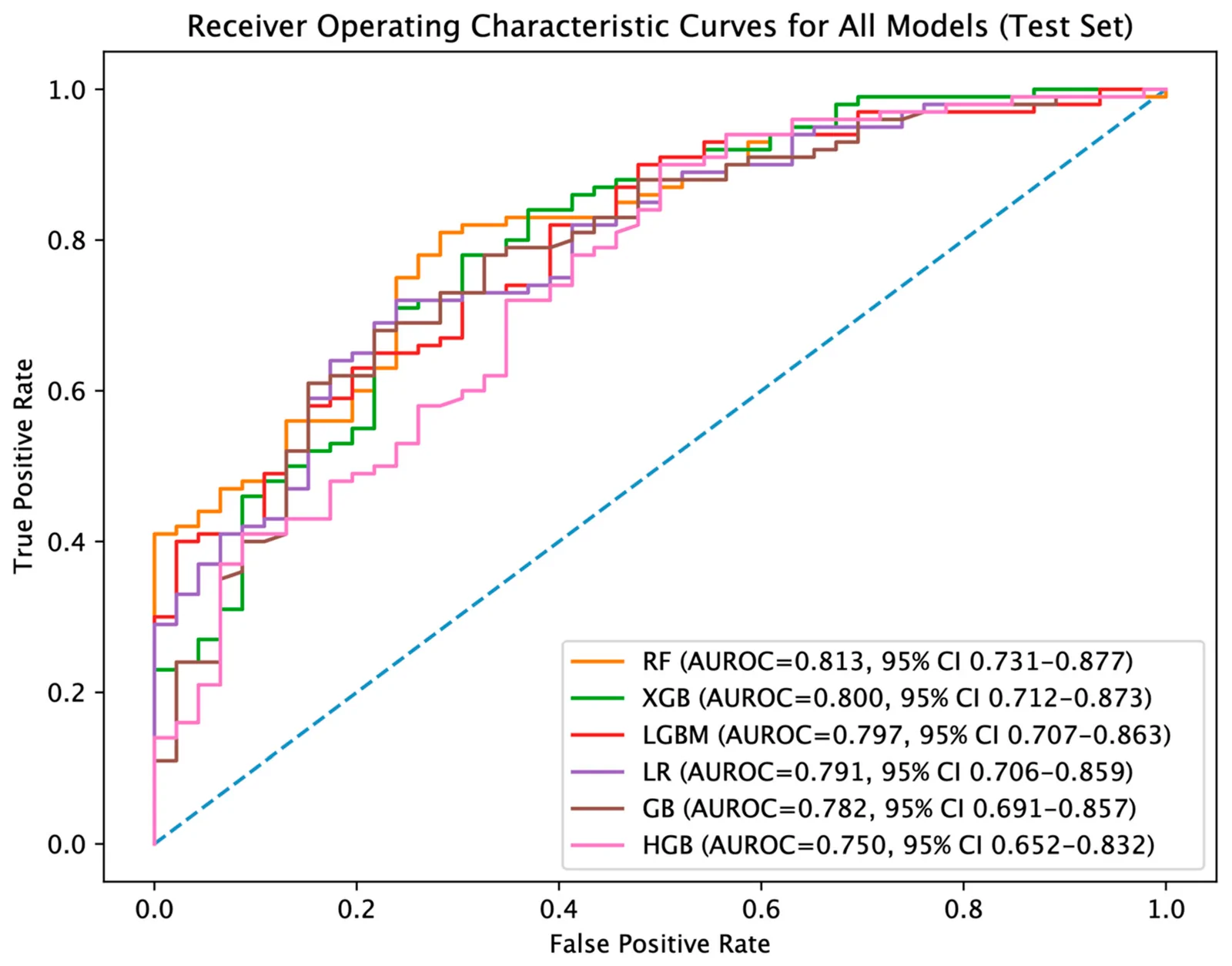
Receiver operating characteristic curves for the machine learning models in the test cohort.

The six candidate models were evaluated on the test cohort, and the AUROCs for each model are shown in Figure 1. The RF model had the highest observed AUROC in the testing cohort (AUROC = 0.813, 95% confidence interval [CI] = 0.731–0.877), followed by the XGB model (AUROC = 0.800, 95% CI = 0.712–0.873) and the LGBM model (AUROC = 0.797, 95% CI = 0.707–0.863). The discrimination and the Brier score for all models are reported in Supplementary Material Table S3a. The results of the sensitivity analysis for tree-based algorithms using the preoperative variables prior to LASSO regularisation did not show improvement in the observed AUROC, as seen in Supplementary Material Table S3b. The cross-validated performance for each model during hyperparameter tuning is reported in Supplementary Material Table S3c. In the RF model, the apparent training AUROC was 0.823 with a mean optimism of 0.122. Therefore, the optimism-corrected training AUROC was 0.701. The DeLong test showed no statistically meaningful difference between the RF and XGB models (z = 0.448, *p* = 0.654).

The calibration of the RF model was evaluated in the independent test cohort. Prior to recalibration, the model demonstrated a Brier score of 0.203 with significant calibration distortion (calibration intercept = 0.62, calibration slope = 3.36), indicating systematic underprediction and under-dispersion of predicted probabilities, with predictions compressed toward the mean. Following logistic recalibration, the Brier score improved to 0.162 and calibration was substantially improved (calibration intercept = 0.06, calibration slope = 0.92). These findings suggest improved overall calibration after recalibration, with minimal residual over-dispersion of predicted probabilities. The pre- and post-recalibration curves are shown in Supplementary Material Figure S4a. Decile-level assessment demonstrated generally close agreement at the lower and upper extremes of predicted probability, with residual local variation in the moderate to high probability range. This variation was not in a consistent direction, where observed TOO rate was lower than predicted in decile 4 (53.3% vs. 60.7% respectively) and decile 7 (64.3% vs. 87.6% respectively), whereas observed TOO rate was higher than predicted in decile 5 (93.3% vs. 71.3%, respectively), as seen in Supplementary Material Table S4b. AUROC was unchanged following logistic recalibration because recalibration preserves the ranking of predicted probabilities.

**Figure 2:**
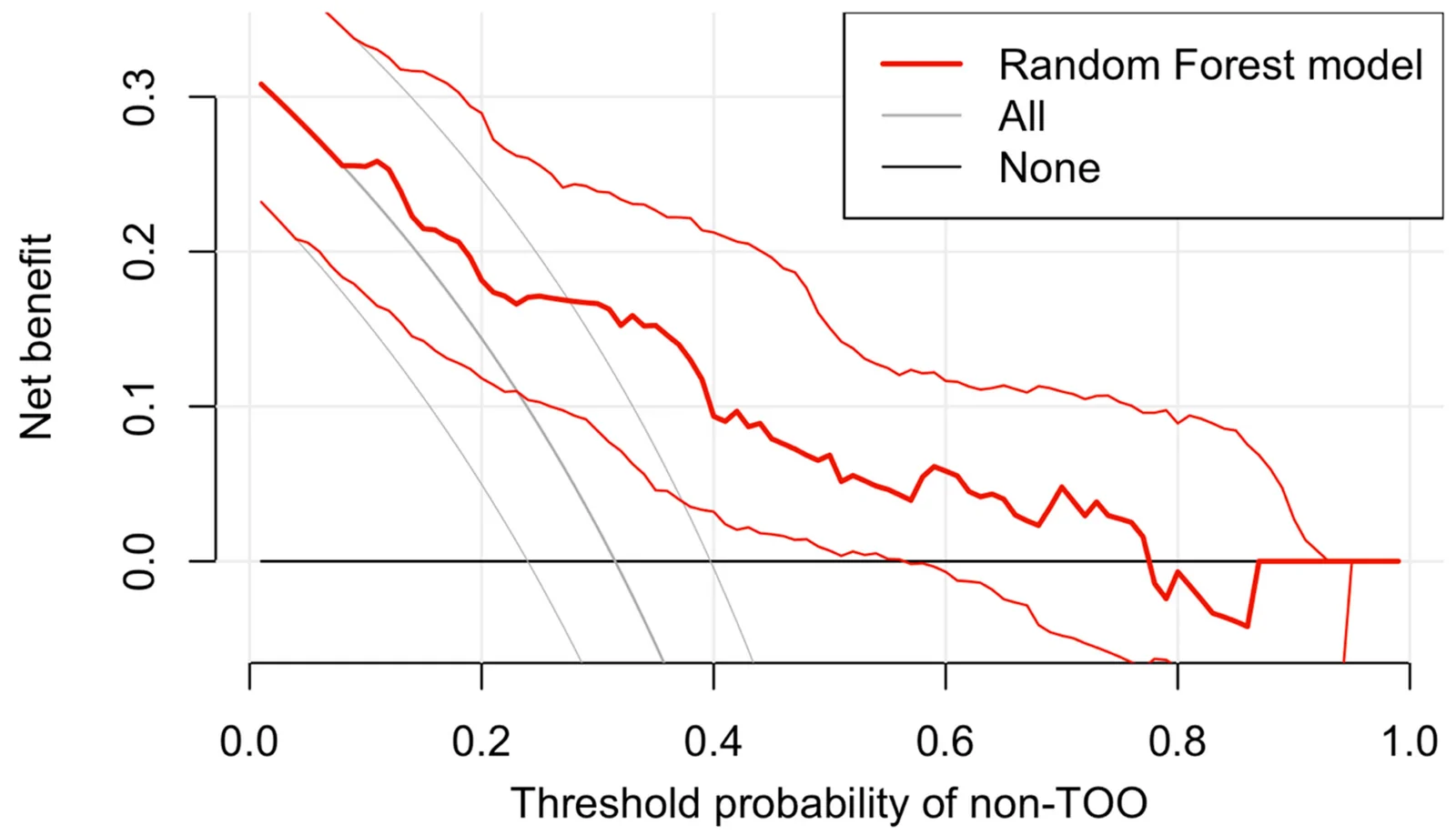
Decision curve analysis for the RF model. The solid red line represents the estimated net benefit of the Random Forest model, with the thinner red lines indicating the corresponding 95% confidence intervals (CIs).

In the exploratory DCA, the RF model demonstrated greater net benefit in both treat-all and treat-none strategies across a broad range of evaluated threshold probabilities for non-achievement of TOO. The greatest separation was observed between approximately 0.1 and 0.7, as seen in Figure 2. As no validated clinical threshold currently exists for predicted non-TOO risk, these findings should not be interpreted as evidence of clinical benefit. At higher threshold probabilities, net benefit approached or fell below the treat-none strategy and became more variable, likely reflecting reduced numbers of patients at the extreme ends of predicted risk. Therefore, estimates in this region should be interpreted with caution.

### 3.2. Impact of Preoperative Variables

**Figure 3:**
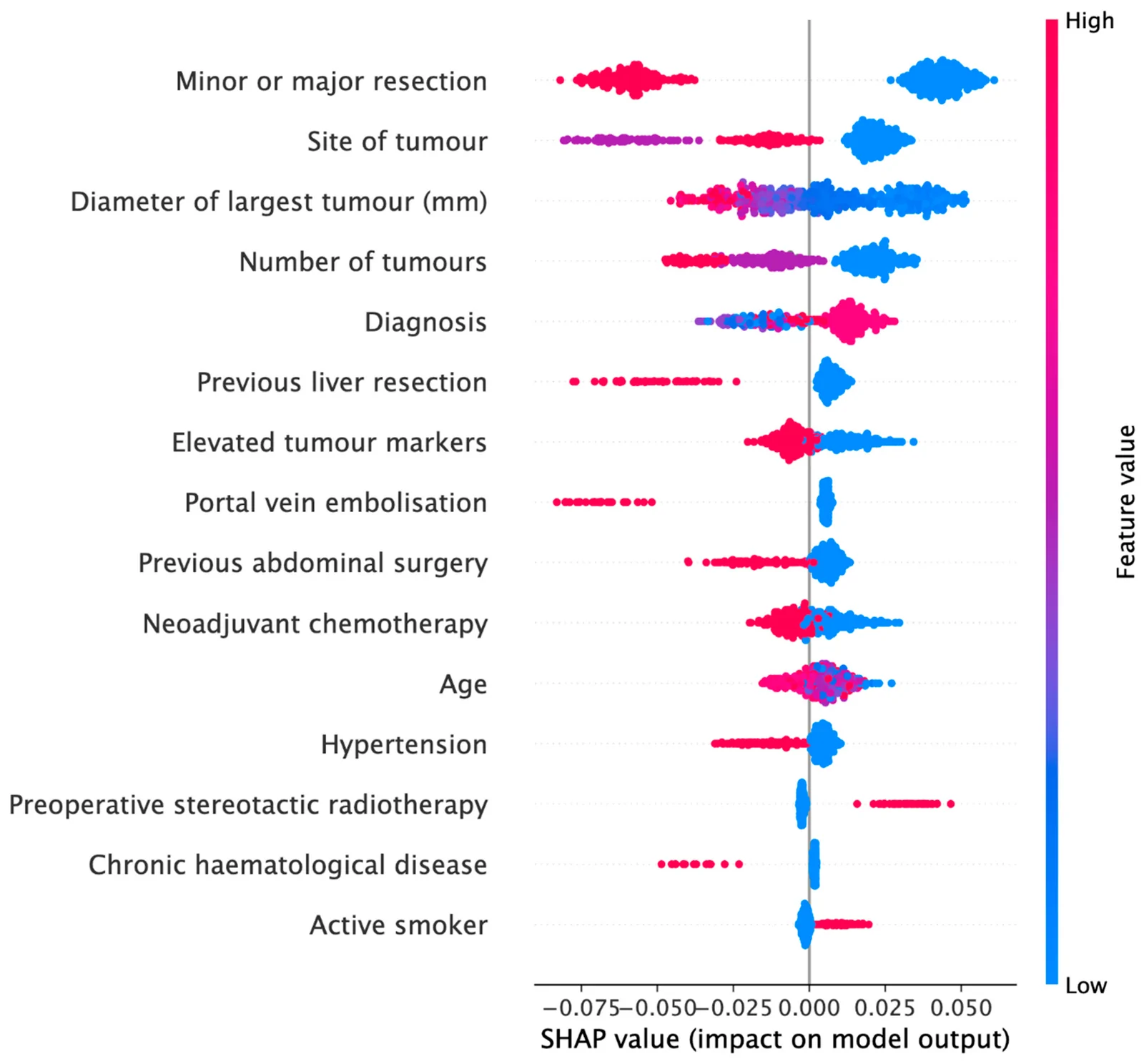
SHAP Beeswarm plot of the top 15 variables included in the RF model.

The top five variables identified as having the largest impact on achieving TOO were the extent of resection (minor vs. major), the site of the tumour (unilateral vs. central vs. bilateral), the diameter of the largest tumour (mm), the number of tumours being resected and the histopathological diagnosis. The SHAP Beeswarm plot for the top 15 variables is shown in Figure 3. The contributions of the variables included in the model are presented in Table 3.

Features contributing toward a higher predicted likelihood of TOO included a minor resection, small tumours, unilateral tumours, no prior liver resection, resecting a solitary tumour, and secondary liver malignancy. Features contributing toward a lower predicted likelihood of TOO included a major resection, large tumours, central or bilateral tumours, having a previous liver resection, having ≥two tumours resected, primary liver malignancy, elevated tumour markers, preoperative portal vein embolisation, neoadjuvant chemotherapy and previous abdominal surgery. Several low-ranking features demonstrated directionally counterintuitive SHAP contributions (chronic illness such as cardiac, respiratory, and renal disease). Given their small mean absolute SHAP values and relatively low prevalence, these findings should not be interpreted as independent protective effects, and may reflect feature correlation, small subgroup sizes or model instability.

### 3.3. Risk Stratification Using Predicted Probability

The cut-offs for the operative groups derived from tertiles of predicted probability of achieving TOO from the training group post-logistic recalibration are shown below:

- Low operative risk: predicted probability ≥ 0.858;
- Moderate operative risk: 0.604 < predicted probability < 0.858;
- High operative risk: predicted probability ≤ 0.604.

The cut-off values for the risk groups pre-recalibration are listed in Supplementary Material S4c. The risk group allocation did not change pre- to post-logistic recalibration.

In the test cohort, the high-operative-risk group had a significantly lower rate of achieving a TOO (37.7%) compared to the low- and moderate-operative-risk groups (90.6%, 80.0% respectively) (*p* < 0.001), as seen in Table 4.

A web-based application has been developed to facilitate the evaluation of the model in external cohorts and prospective validation studies (https://too-risk-calculator.sydney.edu.au/liver-resection-too-predictor/); however, this tool is not ready for clinical practice.

## 4. Discussion

In this study of 729 consecutive patients who underwent liver resection, an ML model was developed to estimate the likelihood of achieving TOO. The RF model demonstrated good discrimination in the internal test cohort (AUROC 0.81). However, lower cross-validated and optimism-corrected performance estimates indicate uncertainty around the expected generalisable performance of the model. There was a marked gradient in observed TOO rates between low- and high-risk groups. However, there was a more modest difference between the low- and moderate-risk groups (90.6% vs. 80.0% respectively), which would be difficult to interpret for clinical decision making. Given that this risk stratification was exploratory and was derived from a single surgeon, further studies should also assess the most clinically meaningful risk stratification and consider other data-driven stratification models. The variables that contributed most strongly to the model predictions were predominantly related to tumour burden and operative complexity, including extent of resection, tumour size and distribution, previous liver resections, tumour number and the diagnosis. Preoperative comorbidities contributed relatively little, which may partly reflect conservative patient selection.

These findings should be interpreted in the context of the composite nature of TOO. In this cohort, the most frequent reason for failure to achieve TOO was an R1/R2 resection margin, followed by major postoperative complications. Accordingly, the model cannot be interpreted solely as a predictor of postoperative recovery. Instead, it appears to capture a combination of oncological burden, intraoperative anatomical and technical complexity, and perioperative risk. This is clinically relevant because patients with large, multiple, centrally located or bilobar tumours, or those requiring a major or repeat liver resections, are also those in whom both margin clearance and uncomplicated recovery may be more difficult to achieve.

The DCA should be interpreted cautiously. The model is not intended to determine whether liver resection should proceed, and a low predicted probability of TOO should not be used as a contraindication to surgery. A more plausible decision pathway is the identification of patients who may benefit from enhanced preoperative review, optimisation and counselling by providing a quantitative appraisal of risk. However, as TOO is a composite endpoint incorporating perioperative outcomes and margin status, the present analysis cannot establish that intervention at any particular risk threshold would improve TOO. Furthermore, no clinically validated threshold exists for escalating care based on predicted TOO risk. The observed net benefit should therefore be regarded as exploratory and requires prospective evaluation using a prespecified intervention and decision threshold.

Moreover, previous studies have demonstrated several similar clinicopathological risk factors for not achieving TOO. In biliary malignancy, a meta-analysis by Yang et al. (2025) identified older age, higher ASA, elevated preoperative bilirubin levels, elevated CA 19-9 levels, large tumour size, an open approach, receiving neoadjuvant therapy, and not receiving adjuvant therapy as being associated with failure to achieve TOO [16]. However, given the retrospective nature of these studies, identifying risk factors such as the operative approach and the need for neoadjuvant treatment should be interpreted cautiously, as straightforward operations were more likely to be completed laparoscopically, and those patients were not likely to have received neoadjuvant treatment. Similar selection effects have been observed in HCC, where higher TOO rates after laparoscopic resection may partly reflect more favourable baseline characteristics rather than a direct effect of operative approach [7]. These findings reinforce the importance of distinguishing underlying tumour and procedural complexity from potentially modifiable perioperative factors.

Computer-based risk prediction tools may support both clinical decision-making and patient communication, with previous studies suggesting potential benefits for preoperative anxiety and patient satisfaction [17]. However, clinical uptake by clinicians is limited by concerns regarding accuracy, accessibility and ease of use, and many models to date have been derived from single-centre cohorts with limited external validation [18,19]. In particular within liver surgery, there has been a concern for combining malignant and benign indications when using risk prediction models, as they underestimate the impact of the malignant process on patient outcomes. This has been demonstrated previously, where generic risk prediction tools such as the ACS-NSQIP surgical risk calculator performed poorly in patients with liver cancer compared to patients with benign conditions [20,21,22]. Given that not all research teams have access to biostatisticians or are familiar with Python, the computer-based web application can be used by most academic clinicians to support further external cohort studies. However, if adequately validated, these tools may complement the MDT decision-making by providing a standardised estimate of operative risk alongside the clinical assessment, particularly when tumour anatomy and technical complexity may not be readily appreciated by all members of the multidisciplinary team.

Several groups have investigated predictive models for TOO in liver surgery. Huang et al. (2025) reported good predictive performance using ML methods in intrahepatic cholangiocarcinoma, although their definition of a TOO differed from the international consensus definition [23]. Wang et al. developed an ML model for benign and malignant indications for liver surgery and reported an AUROC of 0.73 using XGB [24]. Other studies using conventional logistic regression have reported more modest discriminatory performance [25,26]. The current study therefore adds to an evolving literature base by focusing specifically on malignant liver resections and applying the consensus TOO definition. This represents an extension of existing work rather than a fundamentally novel application of ML.

It is worth noting that compared to traditional statistical methods of risk prediction, ML models may offer advantages. In patients who underwent hepatectomy for HCC, Liu et al. (2024) [26] developed and validated a risk score for failure to achieve TOO in a multicentre cohort of 3681 patients. Independent predictors included obesity, ASA score, Child–Pugh grade, tumour size, and the extent of the hepatectomy. The model stratified patients into low-, intermediate- and high-risk groups with good agreement between predicted and observed outcomes in the validation cohort, although the definition for achieving TOO differed from the consensus definition [26]. Similarly, Gundavda et al. (2024) developed an internally validated logistic regression nomogram in 1018 consecutive patients who had a curative liver resections for liver neoplasms at a single centre in India, with an AUROC of 0.75 for predicting TOO [25]. However, that model incorporated perioperative and pathological variables including blood loss and lymphovascular emboli, limiting its use as a purely preoperative prediction tool. These studies demonstrate that conventional statistical models can provide useful risk stratification. In the present study, logistic regression was included as a conventional statistical comparator and demonstrated discrimination broadly comparable to RF (AUROC 0.791 versus 0.813, respectively). Although RF achieved the highest observed AUROC, the absolute difference was modest and does not establish superiority of machine learning over conventional statistical modelling. ML methods may offer additional flexibility by modelling nonlinear relationships and interactions between tumour, anatomical, and procedural factors. We accept that the present study does not establish superiority over conventional statistical modelling, as it has not been tested with a prospectively designed study.

Among the candidate algorithms in the present study, the RF model had the highest discrimination. Calibration was initially distorted but improved after logistic recalibration, with a post-recalibration intercept of 0.06, slope of 0.92 and Brier score of 0.162. Although logistic recalibration improved global calibration measures, residual local variation remained across moderate risk probabilities. Decile-level analysis demonstrated that these deviations occurred in both directions but need to be interpreted in the context of having only 14–15 patients in each decile. The most pronounced overprediction occurred in the seventh decile, highlighting that individual absolute probabilities should not yet be considered sufficiently calibrated for clinical decision-making. The cohort-derived group allocations should therefore be considered descriptive rather than validated clinical thresholds, and external validation with setting-specific recalibration would likely improve the development of risk strata.

The optimal ML approach for clinical risk prediction is uncertain, and many studies compare several algorithms before selecting the model with the best predictive performance [24,27,28,29]. For example, in the model developed by Wang et al. (2025), they similarly evaluated multiple algorithms and selected XGB as the best performing model, with an AUROC of 0.73 [24]. The present study used the same comparative approach, but with RF demonstrating the highest discrimination. The variation in the best-performing algorithms across studies highlights that the model’s performance is data-dependent, and reinforces the need to prioritise reproducibility, calibration and external validation rather than any specific ML method.

The inclusion of intraoperative variables in risk-prediction tools limits the clinical usefulness of these tools for preoperative assessment. Having said that, these variables are important for adjusting outcome data to reflect the technical difficulty of a procedure. The model developed by Gundavda et al. (2024) included the estimated blood loss, and similarly, Ruzzenente et al. (2022) demonstrated that TOO was inversely associated with increased estimated blood loss in addition to operative time [25,30]. This has been demonstrated previously, where the actual operative difficulty was independently associated with lower rates of TOO [12]. Risk prediction has also been shown to improve with the inclusion of intraoperative features, where Xie et al. (2023) demonstrated that a logistic regression risk-prediction model improved performance in predicting TOO with intraoperative variables when compared to only preoperative variables (AUROC 0.762 from 0.698, respectively) [31]. The inclusion of such intraoperative variables obviously means that these models cannot be used for preoperative prediction and patient counselling. Nevertheless, collectively, these findings highlight that the actual technical difficulty of an operation is an important indicator of whether the patient is likely to achieve TOO. Preoperative difficulty estimation tools reasonably differentiate between low, moderate and high operative difficulty groups in achieving TOO [32,33]. Specifically, the IMM score (96%, 86%, and 52% rate of achieving TOO, respectively) and Hasegawa score (99%, 80%, and 64% rate of achieving TOO, respectively) showed the strongest gradient in TOO rates across the difficulty grades [32]. Future studies should aim to assess the discordance between predicted operative difficulty and actual operative difficulty to identify factors that result in improved rates of TOO.

Several limitations should be acknowledged. This was a retrospective, single-surgeon study spanning 24 years, which limits generalisability and introduces potential era effects despite reducing variation in operative technique and decision-making. The majority of procedures were completed as open procedures, which can limit generalisability to modern, minimally invasive practice. The random training/test split therefore represents internal testing, and this model has not been externally validated. There was a small difference in discrimination between the top two models (RF and XGB), and the apparent superiority of RF may therefore reflect sampling variability rather than a meaningful difference in model performance. The low proportion of laparoscopic procedures may limit applicability to contemporary practice, while missing data, particularly for weight, may reflect clinical and documentation-related factors. In addition, variables described as preoperative were only available before surgery, and SHAP values should be interpreted as measures of model contribution rather than independent or causal effect. The substantially higher AUROC observed in the test cohort compared with nested cross-validation and bootstrap optimism-corrected estimates suggests that performance was sensitive to sample partitioning, and that the test-cohort AUROC may represent an optimistic estimate of generalisable discrimination. As R1/R2 margin status was the most common reason for failure to achieve a TOO, the model also appears to reflect disease burden and technical resectability, as well as perioperative risk. Further multicentre and prospective series are required to validate the present findings and permit the deployment of such a tool into clinical practice. Despite these limitations, the present study makes a contribution to the literature by providing a preoperative tool for predicting TOO in liver resection for malignant disease only based on the international consensus definition.

## 5. Conclusions

An ML-based predictive model associated with TOO in patients who underwent liver resection for malignant disease was developed using a large prospectively maintained database. Using the RF algorithm, the model generated risk scores associated with TOO with good internal discriminative performance. This enabled meaningful risk stratification, with a marked gradient across low- to high-operative risk groups in achieving TOO.

If these findings are externally validated, there is potential to use this model for data-driven shared decision making to support preoperative patient counselling and MDT discussions. The integration of such online tools can assist with individualised risk discussions and help with expectation management regarding postoperative care. However, due to the exploratory nature of this study, this model is not ready for widespread clinical use, and further research into the model is warranted.

## Figures and Tables

**Table 1:** Baseline cohort characteristics.

	Total n = 729 (%)	Training n = 583 (%)	Test n = 146 (%)	*p*-Value
Age (mean, SD)	64.5 (11.5)	64.4 (11.7)	65.1 (10.7)	0.497
Gender				0.271
Male	415 (56.9)	326 (55.9)	89 (61.0)	
Female	314 (43.1)	257 (44.1)	57 (39.0)	
Diagnosis				0.697
Colorectal liver metastases	441 (60.5)	353 (60.5)	88 (60.3)	
Hepatocellular carcinoma	88 (12.1)	72 (12.3)	16 (11.0)	
Cholangiocarcinoma	70 (9.6)	52 (8.9)	18 (12.3)	
Gallbladder carcinoma	45 (6.2)	37 (6.3)	8 (5.5)	
Neuroendocrine tumour	25 (3.4)	19 (3.3)	6 (4.1)	
Other liver metastases	58 (8.0)	49 (8.4)	9 (6.2)	
Other malignant	2 (0.3)	1 (0.2)	1 (0.7)	
ASA				0.433
1	292 (40.1)	235 (40.3)	57 (39.0)	
2	276 (37.9)	218 (37.4)	58 (39.7)	
3	151 (20.7)	120 (20.6)	31 (21.2)	
4	10 (1.4)	10 (1.7)	0 (0.0)	
Cardiac disease	230 (31.6)	191 (32.8)	39 (26.7)	0.160
Hypertension	141 (19.3)	117 (20.1)	24 (16.4)	0.321
Ischaemic heart disease	49 (6.7)	36 (6.2)	13 (8.9)	0.239
Atrial fibrillation	31 (4.3)	27 (4.6)	4 (2.7)	0.311
Previous abdominal surgery	170 (23.3)	129 (22.1)	41 (28.1)	0.128
Chronic respiratory disease	74 (10.2)	60 (10.3)	14 (9.6)	0.802
Chronic renal disease	25 (3.4)	20 (3.4)	5 (3.4)	0.997
Chronic kidney disease stage ≥ 3	12 (1.6)			
Single kidney	5 (0.7)			
Chronic thyroid disease	22 (3.0)	19 (3.3)	3 (2.1)	0.447
Other cancer diagnosis	95 (13.0)	78 (13.4)	17 (11.6)	0.578
Hepatitis B/C	47 (6.4)	35 (6.0)	12 (8.2)	0.330
Liver cirrhosis	37 (5.1)	29 (5.0)	8 (5.5)	0.804
Child–Pugh ≥ B	5 (0.7)			
Evidence of chronic liver injury intraoperatively	46 (6.3)			
Active smoker	115 (15.8)	90 (15.4)	25 (17.1)	0.617
EtOH ≥ 100 g/week	177 (24.3)	137 (23.5)	40 (27.4)	0.326
Previous venous thromboembolism	27 (3.7)	19 (3.3)	8 (5.5)	0.204
Diabetes	110 (15.1)	89 (15.3)	21 (14.4)	0.952
Non-insulin dependent diabetes	75 (10.3)	61 (10.5)	14 (9.6)	
Insulin dependent diabetes	35 (4.8)	28 (4.8)	7 (4.8)	
Elevated tumour markers	439 (60.2)	350 (60.0)	89 (61.0)	0.838

**Table 2:** Baseline cohort characteristics continued.

	Total n = 729 (%)	Training n = 583 (%)	Test n = 146 (%)	*p*-Value
Preoperative treatments				
Neoadjuvant chemotherapy	369 (50.6)	303 (52.0)	66 (45.2)	0.144
Stereotactic radiotherapy	58 (8.0)	47 (8.1)	11 (7.5)	0.833
Portal vein embolisation	38 (5.2)	28 (4.8)	10 (6.8)	0.320
Radiofrequency ablation	18 (2.5)	15 (2.6)	3 (2.1)	0.718
Transarterial chemoembolisation (TACE)	12 (1.6)	10 (1.7)	2 (1.4)	0.769
Selective internal radiation therapy (SIR)	11 (1.5)	9 (1.5)	2 (1.4)	0.878
Previous liver resection	76 (10.4)	60 (10.3)	16 (11.0)	0.813
Open resection	653 (89.6)	525 (90.1)	128 (87.7)	0.400
Laparoscopic resection	76 (10.4)	58 (9.9)	18 (12.3)	
Minor resection	439 (60.2)	354 (60.7)	85 (58.2)	0.581
Major resection	290 (39.8)	229 (39.3)	61 (41.8)	
Associated biliary anastomosis	46 (6.3)	37 (6.3)	9 (6.2)	0.935
Associated vascular reconstruction	29 (4.0)	21 (3.6)	8 (5.5)	0.299
Diameter of the largest tumour (mm)—n = 715 (Median, IQR)	38.0 (43.0)	35.0 (49.8) n = 572	40.0 (35.0) n = 143	0.586
Number of tumours				0.112
1	392 (53.8)	322 (55.2)	70 (47.9)	
2–3	219 (30.0)	169 (29.0)	50 (34.2)	
>3	103 (14.1)	83 (14.2)	20 (13.7)	
Missing	15 (2.1)	9 (1.5)	6 (4.1)	
Site of tumour				0.114
Unilateral	470 (64.5)	379 (65.0)	91 (62.3)	
Central	90 (12.4)	77 (13.2)	13 (8.9)	
Bilateral	169 (23.2)	127 (21.8)	42 (28.8)	
Textbook oncological outcome achieved	498 (68.3)	398 (68.3)	100 (68.5)	0.958
Intraoperative adverse event grade ≥ 2	10 (1.4)			
Complication Clavien-Dindo grade ≥ 3a	94 (12.9)			
Mortality within 90 days or within hospital	21 (2.9)			
Postoperative bile leak grade ≥ B	28 (3.8)			
Postoperative liver failure grade ≥ B	27 (3.7)			
90-day readmission for complication Clavien-Dindo grade ≥ 3a	5 (0.7)			
R1 or R2 resection margin	152 (20.9)			

**Table 3:** Directional SHAP impact across variable categories and quartiles (RF model).

Variable	Category/Quartile	Median SHAP (Q1, Q3 †) *	Mean Absolute SHAP Value
Extent of resection	Minor	0.021 (0.018, 0.023)	0.026
	Major	−0.030 (−0.033, −0.028)	0.024
Site of tumour	Unilateral	0.012 (0.010, 0.015)	0.015
	Central	−0.038 (−0.044, −0.034)	0.012
	Bilateral	−0.002 (−0.004, −0.001)	0.001
Diameter of largest tumour	Q1	0.0342	0.035
	Q4	−0.027	0.027
Number of tumours	1	0.015 (0.012, 0.017)	0.015
	2–3	−0.001 (−0.003, 0.000)	0.002
	>3	−0.022 (−0.025, −0.019)	0.007
Diagnosis	HCC	−0.004 (−0.006, −0.001)	0.001
	Cholangiocarcinoma	−0.006 (−0.008, −0.005)	0.002
	Gallbladder cancer	−0.012 (−0.015, −0.010)	0.002
	NET	−0.001 (−0.002, −0.001)	0.000
	CRLM	0.009 (0.007, 0.011)	0.011
	Other liver metastases	0.001 (−0.004, 0.005)	0.001
	Other malignancy	0.000 (0.000, 0.000)	0.000
Previous liver resection	Yes	−0.028 (−0.033, −0.022)	0.006
	No	0.003 (0.002, 0.004)	0.005
Elevated tumour markers	Yes	−0.004 (−0.006, −0.003)	0.006
	No	0.004 (0.001, 0.006)	0.003
Portal vein embolisation	Yes	−0.035 (−0.038, −0.032)	0.004
	No	0.003 (0.002, 0.003)	0.004
Previous abdominal surgery	Yes	−0.008 (−0.012, −0.004)	0.004
	No	0.003 (0.002, 0.004)	0.004
Neoadjuvant chemotherapy	Yes	−0.003 (−0.005, −0.002)	0.004
	No	0.003 (0.001, 0.005)	0.003
Age	Q1	0.007	0.008
	Q4	0.000	0.007
Hypertension	Yes	−0.008 (−0.013, −0.005)	0.004
	No	0.002 (0.001, 0.002)	0.002
Preoperative stereotactic radiotherapy	Yes	0.018 (0.016, 0.022)	0.003
	No	−0.001 (−0.001, −0.001)	0.002
Chronic haematological disease	Yes	−0.021 (−0.024, −0.018)	0.002
	No	0.001 (0.001, 0.001)	0.001
Active smoker	Yes	0.005 (0.003, 0.007)	0.001
	No	−0.001 (−0.001, −0.000)	0.001
Ischaemic heart disease	Yes	0.006 (0.004, 0.007)	0.001
	No	0.000 (0.000, 0.000)	0.001
Atrial fibrillation	Yes	0.005 (0.004, 0.007)	0.001
	No	0.000 (0.000, 0.000)	0.000
Chronic respiratory disease	Yes	−0.004 (−0.005, −0.003)	0.001
	No	0.000 (0.000, 0.001)	0.001
ASA	1–2	0.003 (0.001, 0.004)	0.001
	≥3	−0.001 (−0.001, 0.000)	0.001
Chronic renal disease	Yes	0.003 (0.002, 0.004)	0.000
	No	0.000 (0.000, 0.000)	0.000
EtOH consumption ≥ 100 g/week	Yes	0.002 (−0.001, 0.005)	0.002
	No	0.000 (0.000, 0.001)	0.001
Associated vascular reconstruction	Yes	−0.001 (−0.002, −0.001)	0.000
	No	0.000 (0.000, 0.000)	0.000
Associated biliary anastomosis	Yes	0.001 (−0.002, 0.003)	0.000
	No	−0.000 (−0.001, −0.000)	0.001
Other cancer diagnosis	Yes	0.001 (0.000, 0.001)	0.001
	No	0.000 (0.000, 0.000)	0.001
Cirrhosis	Yes	0.001 (−0.003, 0.003)	0.000
	No	0.000 (0.000, 0.001)	0.001
Previous hepatitis B/C	Yes	0.001 (−0.001, 0.003)	0.000
	No	0.000 (0.000, 0.000)	0.000
Previous venous thromboembolism	Yes	0.000 (−0.002, 0.002)	0.000
	No	0.000 (0.000, 0.000)	0.000

† For categorical variables. * Negative values indicate contribution towards a lower predicted likelihood of TOO and positive values indicate contributions toward a higher predicted likelihood of TOO relative to the model-specific baseline output.

**Table 4:** Chi-square analysis of achieving TOO by operative risk groups.

Group	TOO Achieved (%)	TOO Not Achieved (%)	χ^2^ (Degrees of Freedom)	*p*-Value
Low risk (n = 53)	48 (90.6)	5 (9.4)	37.65 (2)	<0.001
Moderate risk (n = 40)	32 (80.0)	8 (20.0)		
High risk (n = 53)	20 (37.7)	33 (62.3)		

## Data Availability

The data that support the findings of this study are available from the corresponding author, MP, upon reasonable request.

## Supplementary Materials

The following supporting information can be downloaded at: https://www.mdpi.com/article/10.3390/jcm15218527/s1. Table S1: Artificial Intelligence (TRIPOD+AI) checklist.; Table S2a: Definition of included variables; Table S2b: Definition of variables excluded following feature reduction; Table S3a: Model statistics in test cohort; Table S3b: Performance of tree-based models using all eligible variables prior to LASSO regularisation; Table S3c: Nested cross-validated (CV) performance in the training cohort; Figure S4a: Calibration curve for RF model pre- and post-logistic recalibration; Table S4b: Calibration of the RF model in the test cohort following logistic recalibration; Supplementary Material–S4c: Cut-off values for risk groups pre-logistic recalibration.
