# Supplementary material for: Predicting Textbook Oncological Outcomes with Machine Learning: A Retrospective Review of Patients Who Underwent Resection for Malignant Tumours
Source: J Clin Med. 2026 Nov 2;15(21):8527. doi: 10.3390/jcm15218527 (PMC13764353; doi:10.3390/jcm15218527)
Supplement: Supplementary file 1 [file jcm-15-08527-s001.zip › Supplementary Material Table S2.pdf]

Supplementary Material

Supplementary Material - Table S2a: Definition of included variables

The final stable feature set variables:

Variable	Definition
Age	Age of patient (years)
Diameter of largest tumour	Largest cross-sectional tumour diameter (mm)
ASA grade	American Society of Anesthesiologists Physical Status Classification
Diabetes type	Non-diabetic vs non-insulin dependent diabetes vs insulin dependent diabetes
Histopathology	Primary liver cancer (hepatocellular carcinoma, cholangiocarcinoma, gallbladder adenocarcinoma, neuroendocrine tumour) vs secondary liver cancer (metastatic disease from colonic, breast, etc.)
Tumour site	Tumour being resected is unilateral vs central (in segment 4, 5, or 8) vs bilateral (in segment 1, 2, 3 or 4 AND segment 5, 6, 7 or 8)
Extent of resection - Major/Minor classification	Extent of resection by Couinaud classification (1956): minor (≤2 contiguous liver segments) or major (≥3 contiguous liver segments) resection
Operation approach – Open/laparoscopic	Which approach was used for the operation open or laparoscopic
Number of tumours	Number of discrete tumours grouped as 1, 2-3, >3
AF	History of atrial fibrillation
HTN	History of hypertension
IHD	History of ischaemic heart disease
Abdominal surgery	History of previous intra-abdominal surgery
Respiratory	Background of chronic respiratory disease
Renal	Background of chronic renal disease
Other cancers	History of cancer (active or in remission) other than primary diagnosis for procedure
Cirrhosis	Diagnosis of cirrhosis preoperatively
Hepatitis B/C	Previous diagnosis of hepatitis B or C
Haematological	Background of chronic haematological disease
Alcohol (ETOH)	Consumption ≥100g ethanol per week
Smoking	Active smoker within 3 months prior to operation
VTE	History of venous thromboembolic events
Elevated tumour markers	Elevated tumour markers for the respective malignancy being resected (e.g., AFP for HCC, CA19-9 for cholangiocarcinoma, CEA for colorectal metastases, etc)
Preoperative neoadjuvant chemotherapy to the liver	Whether patient received neoadjuvant chemotherapy prior to surgery
Stereotactic radiotherapy	Whether patient underwent stereotactic radiotherapy prior to surgery
Portal vein embolisation	Whether patient underwent portal vein embolisation prior to surgery
Previous liver resection	History of previous liver resection

Associated vascular resection	Whether there was a planned requirement for vascular resection
Associated biliary anastomosis	Whether there was a planned requirement for biliary anastomosis

Supplementary Material - Table S2b: Definition of variables excluded following feature reduction

Variable	Definition	Reason for exclusion
CHF	History of congestive heart failure	Constant feature, single value in 99.9% of all patients.
Cirrhosis – Child Pugh ≥B	Presence of cirrhosis with Child-Pugh grade ≥B	Constant feature, single value in 99.3% of all patients.
Microwave ablation	Treatment for liver cancer with microwave ablation preoperatively	Constant feature, single value in 99.2% of all patients.
Dermatological	Pre-existing dermatological condition	Constant feature, single value in 98.9% of all patients.
Selective internal radiation therapy	Treatment for liver cancer with selective internal radiation therapy preoperatively	Constant feature, single value in 98.5% of all patients.
Chronic kidney disease	Presence of chronic kidney disease	Constant feature, single value in 98.4% of all patients.
OSA	Presence of obstructive sleep apnoea	Constant feature, single value in 98.2% of all patients.
COPD	Presence of chronic obstructive pulmonary disease	Constant feature, single value in 98.1% of all patients.
Dyspnoea	Presence of dyspnoea preoperatively	Constant feature, single value in 97.9% of all patients.
RFA	Treatment for liver cancer with radiofrequency ablation preoperatively	Constant feature, single value in 97.5% of all patients.
Urological	History of urological comorbidities	Constant feature, single value in 97.5% of all patients.
Cardiac co-morbidity	Any pre-existing cardiac disease	Excluded following LASSO regularisation
Liver disease	History of any chronic liver disease	Excluded following LASSO regularisation
Endocrine disease	Background of chronic endocrine disease	Excluded following LASSO regularisation

Family history of malignancy	Any family history of malignancy	Excluded following LASSO regularisation
Gastrointestinal disease	Any pre-existing gastrointestinal disease	Excluded following LASSO regularisation
Musculoskeletal	History of chronic musculoskeletal disease	Excluded following LASSO regularisation

Supplementary Material - Table S3a: Model statistics in test cohort

Model	AUROC (95% Confidence interval)	Brier score pre-recalibration	Brier score post-recalibration
RF	0.813 (0.731-0.877)	0.203	0.162
XGB	0.800 (0.712-0.873)	0.235	0.172
LGBM	0.797 (0.707-0.863)	0.236	0.165
LR	0.791 (0.706-0.859)	0.219	0.170
GB	0.782 (0.691-0.857)	0.199	0.171
HGB	0.750 (0.652-0.832)	0.187	0.179

Supplementary Material - Table S3b: Performance of tree-based models using all eligible variables prior to LASSO regularisation

Model	AUROC (95% Confidence interval)	Brier score
RF	0.780 (0.702 – 0.854)	0.214
XGB	0.801 (0.721 – 0.872)	0.235
LGBM	0.799 (0.725 – 0.869)	0.237

Supplementary Material - Table S3c: Nested cross-validated (CV) performance in the training cohort

Model	Nested CV AUROC, mean ± SD
RF	0.608 ± 0.029
XGB	0.606 ± 0.010
LGBM	0.610 ± 0.019
LR	0.637 ± 0.016
GB	0.601 ± 0.013
HGB	0.577 ± 0.013

Supplementary Material – Figure S4a: Calibration curve for RF model pre- and post-logistic recalibration

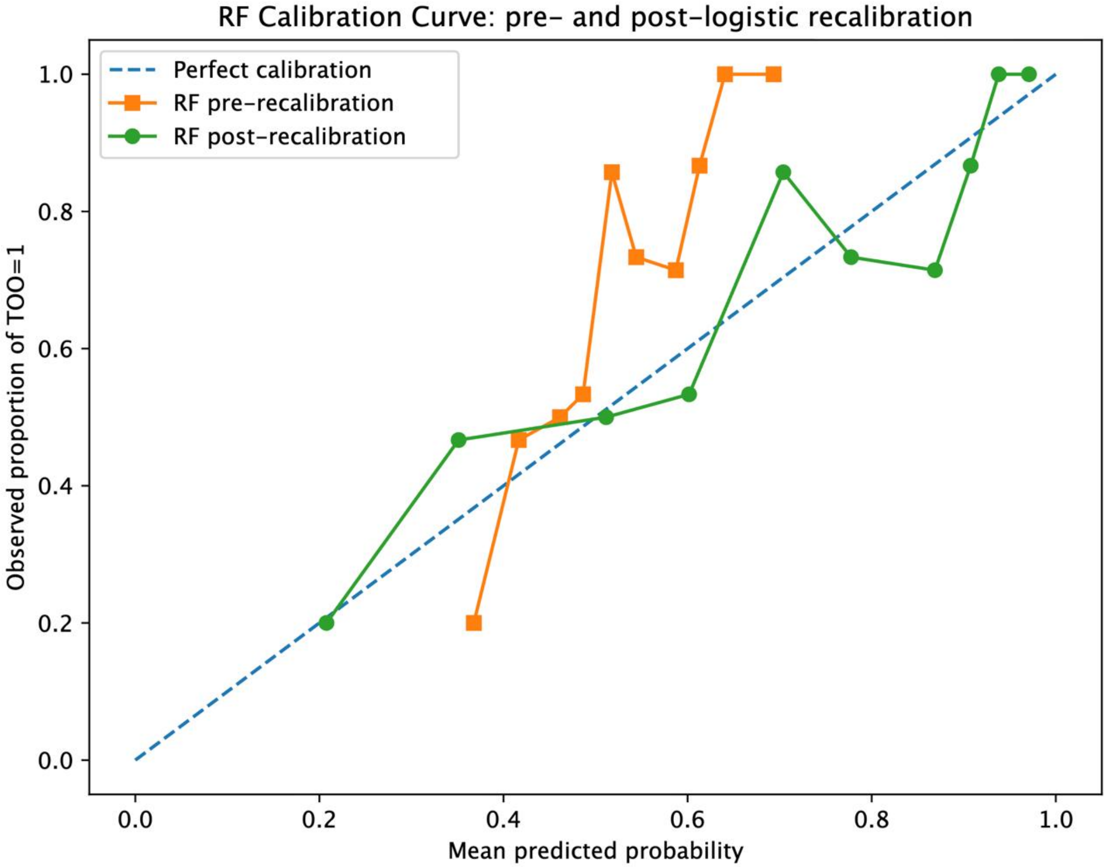

Supplementary Material – Table S4b: Calibration of the RF model in the test cohort following logistic recalibration

Decile	n	Predicted TOO probability range	Mean probability of TOO	Observed TOO, n (%)
1	15	0.089 - 0.277	0.208	3 (20.0)
2	15	0.285 - 0.441	0.351	7 (46.7)
3	15	0.442 - 0.568	0.515	7 (46.7)
4	15	0.573 - 0.643	0.607	8 (53.3)
5	15	0.662 - 0.744	0.713	14 (93.3)
6	15	0.754 - 0.849	0.791	11 (73.3)
7	14	0.850 - 0.895	0.876	9 (64.3)
8	14	0.899 - 0.927	0.911	13 (92.9)
9	14	0.931 - 0.949	0.940	14 (100.0)
10	14	0.952 - 0.990	0.972	14 (100.0)

Supplementary Material – S4c: Cut-off values for risk groups pre-logistic recalibration

The cut-offs for the operative groups derived from tertiles of predicted probability of achieving TOO from the training group pre-logistic recalibration are shown below:

- Low operative risk: predicted probability ≥ 0.580
- Moderate operative risk: 0.487 < predicted probability < 0.580
- High operative risk: predicted probability ≤0.487
